# Understanding the Role of the Antioxidant Drug Erdosteine and Its Active Metabolite on *Staphylococcus aureus* Methicillin Resistant Biofilm Formation

**DOI:** 10.3390/antiox10121922

**Published:** 2021-11-29

**Authors:** Cristina Cattò, Federica Villa, Francesca Cappitelli

**Affiliations:** Department of Food, Environmental and Nutritional Sciences, Università degli Studi di Milano, 20122 Milano, Italy; federica.villa@unimi.it (F.V.); francesca.cappitelli@unimi.it (F.C.)

**Keywords:** erdosteine, metabolite I, biofilm, antioxidant, oxidative stress, nitric oxide, *S. aureus*, proteomics

## Abstract

Increasing numbers of researches have suggested that some drugs with reactive oxygen species (ROS)-mediated mechanisms of action modulate biofilm formation of some pathogenic strains. However, the full contribution of ROS to biofilm development is still an open question. In this paper, the correlations between the antioxidant drug Erdosteine (Er) and its active Metabolite I (Met I), ROS and biofilm development of two strains of methicillin resistant *Staphylococcus aureus* are presented. Experiments revealed that Er and Met I at 2 and 5 mg/L increased up to three orders of magnitude the number of biofilm-dwelling cells, while the content of ROS within the biofilms was reduced above the 87%, with a major effect of Met I in comparison to Er. Comparative proteomics showed that, 5 mg/L Met I modified the expression of 30% and 65% of total proteins in the two strains respectively. Some proteins involved in cell replication were upregulated, and a nitric oxide-based mechanism is assumed to modulate the biofilm development by changing quorum sensitive pathways. Additionally, several proteins involved in virulence were downregulated in the presence of Met I, suggesting that treated cells, despite being greater in number, might have lost part of their virulence.

## 1. Introduction

Despite improvements in prevention, biofilm-related infections remain a significant clinical problem as bacteria in the form of biofilms display high tolerance to antibiotics and easily evade the patient’s immune system [1]. Additionally, resistance of many pathogenic microorganisms toward antibiotics is an emerging challenge in the medical sector [2]. As a consequence, biofilm related infections are challenging or even impossible to treat and expose patients to infection persistence and recurrences with drastic medical complications, including mortality, and relevant financial burdens [3].

An increased number of reports has suggested that reactive oxygen species (ROS) might have a regulatory function in biofilm development as signalling molecules [4]. Indeed, growing evidence shows that some drugs currently used to treat critically ill patients, e.g., cancer chemotherapies, mucolytics or antibiotics, at certain concentrations, modulate biofilm formation of some pathogenic strains, inhibiting or even promoting their growth [5,6]. Interestingly, the mode of action of these drugs is accompanied by the generation or depletion of ROS, further supporting the theory that ROS may effectively trigger the biofilm response upon drug exposure.

**Figure 1:**
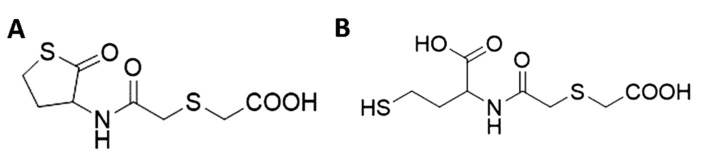
Chemical structure of the antioxidant drug Er (*N*-(carboxymethylthioacetyl)-homocysteine thiolactone) (**A**) and Met I ((±)-*N*-(2-carboxymethylthioacetyl)homocysteine) (**B**).

For example, salicylic acid, the active component of aspirin, has been proved to interact with the quinone oxidoreductase WrbA, reducing *Escherichia coli* biofilm through a mechanism that depends on ROS [5,7]. N-acetylcysteine is used as mucolytic drug and it is applied on a broad range of pathologies with the primary role of antioxidant agent [8]. *N*-acetylcysteine has been described as both decreasing or increasing the biofilm formation by different bacterial strains, depending on the experimental conditions [9,10,11,12,13,14,15]. Further, 3-bromopyruvate and 5-fluorouracil are anti-cancer drugs that act by inducing oxidative stress via depletion of antioxidants and inactivation of antioxidant enzymes [16,17]. Interestingly, 3-bromopyruvate was found able to disrupt pre-formed *Staphylococcus aureus* biofilms [18] whereas 5-fluorouracil is a potent quorum-quencher [19]. However, the exact contribution of ROS to biofilm formation and the correlation between the administration of these drugs with a ROS mediated mechanism of action and biofilm development is still an open question [20]. Medical doctors should be aware that that some antioxidant-based drugs, at certain concentrations and under specific conditions, may promote the growth of pathogenic biofilms, complicating the already compromised patient’s conditions. In this study, the role of the antioxidant drug Erdosteine (Er, *N*-(carboxymethylthioacetyl)-homocysteine thiolactone, Figure 1A) in biofilm formation and its involvement in the cellular redox balance was thoroughly investigated using Methicillin-resistant *S. aureus* (MRSA) as a model system.

Er is rapidly absorbed after oral administration. This drug contains two blocked -SH groups, one of which is a thioether in the aliphatic side chain and the other is enclosed in the heterocyclic ring (thiolactone). In the acidic media of the stomach the molecule is stable. When the drug is exposed to a more alkaline pH, the thiolactone ring opens, generating in the bloodstream the metabolite (±)-*N*-(2-carboxymethylthioacetyl)homocysteine (Met I, Figure 1B) that has a free thiol group available [21]. The pharmacological and clinical effects of Er are mainly due to the activity of Met I. The liberated sulfhydryl groups break the disulphide bonds of the bronchial mucoproteins, which hold the glycoprotein fibres of mucus together. This makes the bronchial secretions more fluid, modulating mucus production and viscosity, and increasing mucociliary transport, thereby improving expectoration [22]. Er can also act as antioxidant drugs by offering free -SH groups as a source of reducing equivalents, and by replenishing intracellular glutathione (GSH) levels. Moreover, Er interferes with inflammatory pathways and can modulate the tone of airway smooth muscle in human bronchi [23]. No accumulation or change in the metabolism of Er and Met I have been observed after oral administration of 600 to 900 mg daily for 8 days. Er is mainly excreted in the urine as an inorganic sulfate [24]. Thus, Er is a homocysteine-derived thiol derivative with mucolytic and free radical scavenging properties approved for the clinical management of several respiratory diseases where the overlap of bacterial infection is present [23].

Scientific evidence showed that thiol-based drugs can reduce bacterial adhesion to the respiratory epithelial cell surface, inhibiting biofilm formation and improving the efficacy of antibiotic therapy [25]. It has been reported that 2.5, 5 and 10 mg/L of Met I acts at a fimbrial level, by reducing both *S. aureus* and *Escherichia coli* adhesiveness to human mucosal epithelial cells that is the first step in the development of respiratory tract infections [26]. Braga et al. [26] suggested that Er and Met I exert their anti-adhesion activity by inducing a stereochemical conformational change of the pilin molecules that impedes their binding to the receptors of human cells. However, they neither verified whether the resulting data came from a biocidal activity of the drug rather than a real anti-adhesion effect, nor gave proofs about the drug’s activity on the pilin molecules. Therefore, the exact Er and Met I mechanisms of action and the nature of their targets has remained unknown. No less importantly, these researches have been limited to the first step of biofilm development, i.e., the adhesion step, employing simple systems based on static and batch-growth conditions that hardly reproduce the complexity of biofilms in the real environments [27]. Thus, the effect of Met I on biofilm development is still a crucial question that remains to be addressed to better understand the role of antioxidants on biofilm development and to determine which of the pharmacological effects contribute to the clinical benefits observed using this thiol-based compound.

Therefore, in order to better simulate in vivo conditions, the research was concentrated on biofilm response upon Met I treatment and Er was analysed in comparison. Moreover, a label-free quantitative proteomic approach was applied to explain the distinct protein expression patterns between untreated and treated biofilms to reveal potential determinants associated with the thiol-based drug.

## 2. Materials and Methods

### 2.1. Staphylococcus aureus Strains and Growth Conditions

Methicillin-resistant *S. aureus* (MRSA) strains ATCC 43300 and 98825 TX (isolated from human hospital patients) were used as a model system for bacterial sessile growth. The strains were stored at −80 °C in suspensions with 20% glycerol and 2% peptone, and were grown in Tryptic Soy Broth (TSB, Sigma-Adrich, St. Louis, MO, USA) at 37 °C.

### 2.2. MRSA Planktonic Growth

Bacterial growth with Er and Met I (Recipharm, Stockholm, Sweden) at 2 and 5 mg/L was determined according to Cattò et al. [28]. Briefly, planktonic growth assays were performed in TSB medium supplemented with 2 and 5 mg/L of each drug in 96-well microtiter plates. Negative control wells are left unpopulated and untreated, to calibrate the base level of machine output if all cells were to die. Positive control wells are left untreated as to measure the base response for drugs that have no effect [29]. Cells of an overnight culture were added to obtain a final concentration of 10^7^ cells/mL and grown at 37 °C. Growth was measured reading the optical density at 600 nm (OD_600_) every 15 min for over 36 h using the Infinite 200 PRO Microplate Reader (Tecan, Männedorf, Switzerland). Kinetics were obtained by plotting the A600 of suspensions minus the A600 of the non-inoculated broth against incubation time. The polynomial Gompertz model was used to fit the growth curves, and the maximum specific growth rate (µ_m_) and the maximum growth (Y_M_) were calculated using GraphPad Prism software (version 5.0, San Diego, CA, USA). Four biological replicates were carried out for each treatment. The experiment was repeated at least four times.

### 2.3. Biofilm Growth in the CDC Reactor

MRSA biofilms were grown without and with Er and Met I at 2 and 5 mg/L in the Center for Disease Control biofilm reactor (CDC reactor, Biosurface Technologies, Bozeman, MT, USA) according to Cattò et al. [5]. The positive controls were the bacterial biofilms without the addition of any drugs. The bioreactor was inoculated with 400 mL of sterile TSB broth with the addition of 1 mL of diluted pre-washed overnight culture with 10^7^ cells of each MRSA strains. The culture was grown at 37 °C with continuous stirring for 24 h. When the 24-h adhesion phase was ended, the peristaltic pump was started and sterile 5% TSB broth was pumped into the reactor at a rate of 8.3 mL/min. After 48 h of the dynamic phase, coupons (polycarbonate round surfaces) were removed from the bioreactor, gently washed with phosphate buffered saline (PBS, 0.01 M phosphate buffer, 0.0027 M potassium chloride pH 7.4) and the biomass was analysed.

### 2.4. Biofilm-Dwelling Cells

Collected coupons were suspended in 2 mL of PBS and biofilm cells were dislodged from the coupon surface by 30 s vortex mixing and 2 min sonication bath (Branson 3510, Branson Ultrasonic Corporation, Dunburry, CT, USA) followed by other 30 s vortex mixing. Serial dilutions of the resulting cell suspensions were plated on Tryptic Soy Agar (TSA, Sigma-Aldrich, St. Louis, MO, USA) and incubated overnight at 37 °C. The standard colony counting method was applied to count colony forming units (CFUs). The numbers of viable bacterial cells normalized to the area were reported and means were calculated. The effect of Er and its metabolite against biofilm formation was calculated as reduction/increase of the CFU/cm^2^ in comparison to the positive control. The experiment was repeated three times and three coupons were analyzed in each experiment.

### 2.5. Biofilm Morphology

Three-D morphology of biofilms grown without and with Er and its Met I at 5 mg/L were analysed by CLSM according to Cattò et al. [30]. Biofilms were stained with 1:1000 SYTO 9 fluorescent nucleic acid dye (excitation 485 nm/498 nm, ThermoFisher, Milan, Italy) and lectin Concanavalin A-Texas Red conjugate (ConA, Thermo Fisher Scientific, Waltham, MA, USA) in the dark for 30 min to display sessile-dwelling cells and the polysaccharide component of the biofilm matrix, respectively. Confocal images were collected using a Nikon A1 laser scanning confocal microscope and a 20× or 40× objectives. The 488 nm and 561 nm laser lines and the 500–550 nm and 590–630 nm emission filters were used to visualize the green signal from the biofilm-dwelling cells and the biofilm matrix, respectively. Captured images were analysed with the software NIS-Elements by Nikon (Nikon, Amsterdam, The Netherlands) for the 3D reconstructions of the biofilms. Three coupons for each treatment were analysed and 3 random positions for each coupon were observed.

### 2.6. Oxidative Stress Level

Collected coupons were suspended 2 mL of PBS and biofilm was removed from the coupon surface by vortex mixing and sonication as reported above for the cell abundance experiment. Biofilms were broken by sonication (five 1-min sonication cycles at 22 μm amplitude followed by 1-min cooling periods, in Soniprep 150) and the level of oxidative stress was evaluated using the 2′,7′-dichlorodihydrofluorescein diacetate (H_2_DCFDA) assay as by Jakubowski and colleagues [31]. In brief, 5 mM H_2_DCFDA was supplemented to the sample to a final concentration of 10 μM and samples were incubated at 30 °C. After 30 min, the samples were centrifugated and fluorescence of the supernatant was evaluated using the Infinite 200 PRO Microplate Reader (Tecan, Männedorf, Switzerland) with excitation at 488 nm and emission at 520 nm. Fluorescence data were normalized to the number of cells per cm^2^ and means were calculated. Biofilms from three coupons were analysed for each treatment. The experiment was repeated four times and three coupons were analyzed in each experiment.

### 2.7. Biofilm Dispersion

To evaluate the strength of biofilm to surface detachment after drug treatments, coupons with biofilm pre-grown without and with Er and its Met I at 2 and 5 mg/L were removed from the CDC reactor and soaked in PBS. After 1 h, biofilms were dislodged from the coupon surface according to the previous section and viable cells were assessed by plate count viability assay. Cells in the bulk PBS were also collected and quantified by plate count viability assay. The tendency of biofilm detachment was calculated as: (no. of viable cells from bulk PBS × 100)/(no. of viable cells from bulk PBS + no. of viable cells from the coupon biofilm) and means were calculated. Three biological replicates were carried out for each treatment. The experiment was repeated three times.

### 2.8. Statistical Analysis

Two-tailed ANOVA analysis (GraphPad Prism Software, version 8.0.0, San Diego, CA, USA) was applied to study statistically significant differences among the biofilm samples and concentrations. The ANOVA analysis was performed after verifying data independence (Pearson’s Chi-square test), normal distribution (D’Agostino-Pearson normality test) and homogeneity of variances (Bartlett’s test). Tukey’s honestly significant difference test (HSD) was employed for pairwise comparisons to determine the data significance. Statistically significant findings were assessed by *p*-values < 0.05.

### 2.9. Protein Extraction and Sample Preparation

Biofilms grown without and with Met I at 5 mg/L were scraped from 6 coupons and suspended in 6 M urea to a final concentration of 125 μg/mL. The suspension was vortexed for 15 min, incubated overnight at room temperature and centrifuged for 30 min at 11,000× *g*. The amount of proteins was determined by the Bradford assay following the standard procedure.

An appropriate quantity of proteins in 50 mM NH₄HCO₃ were reduced with dithiothreitol (5 mM final concentration, Sigma-Aldrich, St. Louis, MO, USA) for 30 min at 55 °C. Iodoacetamide (Sigma-Aldrich, St. Louis, MO, USA) was added to a final concentration of 15 mM and samples were incubated for 30 min in the dark at room temperature. Proteins were digested with trypsin (Promega Italia SRL, Milano, Italy) using an enzyme–protein ratio of 1:20 at 37 °C overnight and the reaction was terminated by adding trifluoroacetic acid (Sigma-Aldrich St. Louis, MO, USA,, final concentration of 0.5%). To improve the quality of instrumental analysis, the digested samples were further purified and concentrated using a 0.2 μL C-18 resin ZipTip (Millipore, Burlington, VT, USA).

### 2.10. High-Resolution Mass Spectrometry Analysis (nLC-HRMS)

One µg total of sample was analysed by a Dionex Ultimate 3000 nano-LC system (Sunnyvale, CA, USA) combined with an Orbitrap Fusion™ Tribrid™ Mass Spectrometer (Thermo Scientific, Bremen, Germany) equipped with a nano electrospray ion source. Peptide mixtures were pre-concentrated onto an Acclaim PepMap 100—100 μm × 2 cm C18 (Thermo Fisher Scientific, Waltham, MA, USA) and separated on EASY-Spray column, 25 cm × 75 μm ID packed with Thermo Scientific Acclaim PepMap RSLC C18, 3 μm, 100 Å, at 35 °C and flow rate 300 nL/min. Mobile phases were the following: 0.1% formic acid (FA) in water (Buffer A); 0.1% FA in water/acetonitrile with 2/8 ratio (Buffer B). The elution gradient was from 96% Buffer A to 95% Buffer B for 110 min. MS spectra were collected over an m/z range of 375–1500 Da at 120,000 resolutions, operating in the data dependent mode, cycle time 3 s between master scans. Higher-energy collision dissociation was carried out with collision energy set at 35 eV and a positive polarity. The experiment was repeated three times and each sample was run in three technical replicates.

### 2.11. Bioinformatic, Statistical and Functional Annotation Analysis

Raw data were analyzed against a protein database using SEQUEST algorithm in Proteome Discoverer software version 2.2 (Thermo Fisher Scientific, Scientific, Waltham, MA, USA) for peptide/protein identification. The searches were made against Uniprot KnowledgeBase (KB)/Swiss-Prot (taxonomy ID 1280—*Staphylococcus aureus*). The minimum peptide length was 6 amino acids and enzymatic digestion with trypsin was used, with maximum 2 missed cleavages. A precursor mass tolerance of 8 ppm and fragment mass tolerance of 0.02 Da were employed; Met loss + acetylation (N-term) and oxidation (M) were used as dynamic modifications and carbamidomethylation (C) as static modification.

A decoy database search was used to obtain the peptide false discovery rate with percolator module. The false discovery rates at the protein and peptide level were 0.01 for highly confident peptide-spectrum matches and 0.05 for peptide-spectrum matches with moderate confidence respectively.

Protein quantification was based on the label-free quantification (LFQ). The mean LFQ intensities and the standard deviation of this value were determined for all experimental groups. The fold changes in the level of the proteins were assessed by comparing the mean LFQ intensities among all experimental groups. A protein was considered differentially expressed if the difference was statistically significant (*p* < 0.001), the fold change minimum was ±1.5, and it was identified with a minimum of two peptides.

The fold changes were transformed using the log_2_ function while the *p* value was log_10_ transformed for Volcano plot scaling using GraphPad Prism (GraphPad Prism Software, version 8.0.0, San Diego, CA, USA).

Differentially abundant proteins were subjected to GO classification via the UniProt Classification System database [32] to study biological processes, molecular function and cellular compartment.

## 3. Results

### 3.1. Erdosteine and Metabolite I Do Not Affect MRSA Planktonic Growth

In Supplementary Materials Figure S1A,B planktonic growth curves of MRSA strains grown with Er and its Met at 0, 2 and 5 mg/L were reported. No significant differences between the treated samples and the positive controls were obtained in both ATCC 43300 and 98825 TX strains. No significant reduction in the maximum specific growth rate and in the maximum growth were observed among samples (Supplementary Materials Figure S1C). 

### 3.2. Erdosteine and Metabolite I Promote Biofilm Formation

**Figure 2:**
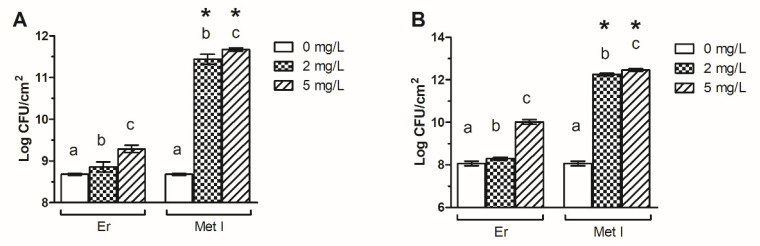
Adhered cells (CFU/cm^2^) within MRSA ATCC 43300 (**A**) and 98825 TX (**B**) biofilms grown with Er and Met I at 0, 2 and 5 mg/L. Data report the mean ± standard deviation of three independent values. Different superscript letters indicate significant differences (Tukey’s HSD, *p* ≤ 0.05) between the means of different concentrations whereas a * indicates significative differences between Er and the corresponding counterpart grown with Met I at the same concentration.

Experiments revealed that both Er and Met I increased the number of adhered cells within the biofilms and the effect was major upon Met I treatment in comparison to Er (Figure 2 and Supplementary Materials Table S1).

The 2 mg/L Er increased the number of cells of ATCC 43300 and 98825 TX strains up to 73.0% and 67.0% respectively, whereas 5 mg/L Er enhanced the biofilm cellular amount by one order of magnitude in both biofilms. Further, 2 and 5 mg/L Met I increased the number of ATCC 43300 biofilm cells by one or two orders of magnitude respectively, and by three orders of magnitude in 98825 TX biofilm treated with both concentrations.

**Figure 3:**
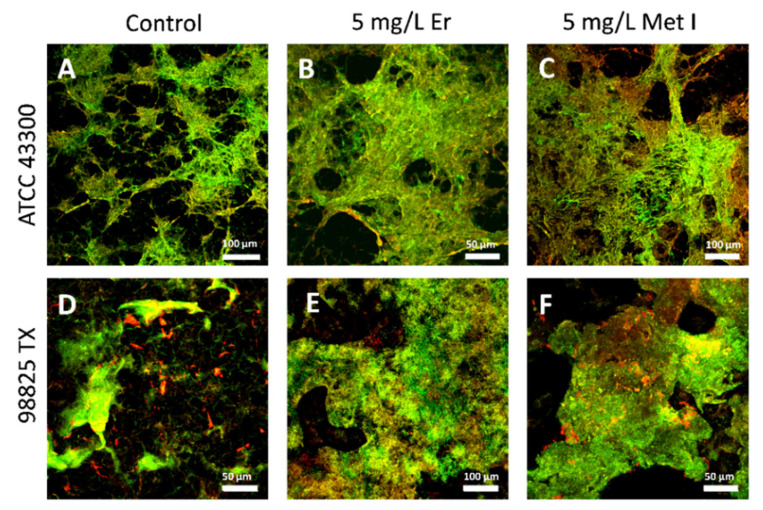
Representative views of 3-D CLSM images of biofilm developed without (**A**,**D**) and with Er (**B**,**E**) and Met I (**C**,**F**) at 5 mg/L. Live bacteria were stained green with SYTO 9 fluorescent nucleic acid dye while the biofilm matrix was visualized in red by the lectin Concanavalin A stain. Scale bar = 50 or 100 μm.

These results were corroborated by microscopic observation. Representative biofilm structures under various treatments are reported in Figure 3, Supplementary Materials Figure S2 and S3. The presence of the matrix confirmed the biofilm growth for both the strains at laboratory scale. Control samples presented scattered cells and some small bacterial clusters. After treatments, the biofilm biomass increased, forming interconnected clusters with cells oriented horizontally and a few scattered cells were observed.

### 3.3. Erdosteine and Metabolite I Reduce the Oxidative Stress Level within the Biofilm

**Figure 4:**
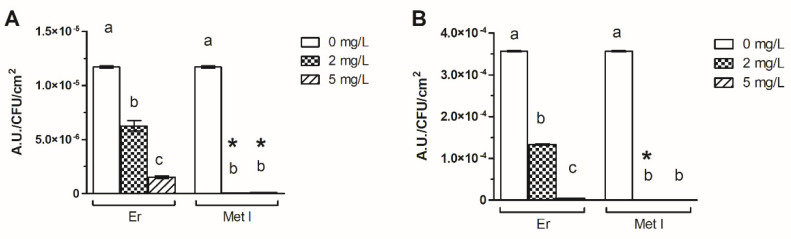
Level of oxidative stress within the MRSA ATCC 43300 (**A**) and 98825 TX (**B**) biofilms grown with Er and Met I at 0, 2 and 5 mg/L. Data report the mean ± standard deviation of three independent measurements. Different superscript letters indicate significant differences (Tukey’s HSD, *p* ≤ 0.05) between the means of different concentrations whereas a star indicates significative differences between Er and the corresponding counterpart grown with Met I at the same concentration.

Experiments revealed that Er and Met I decreased the oxidative stress within the biofilms of both MRSA ATCC 43300 and 98825 TX strains (Figure 4 and Supplementary Materials Table S2). Met I displayed a major effect in comparison to Er as it decreased the level of oxidative stress in the biofilm above 99% with comparable results for both strains and concentrations. A similar effect was found when the 98825 TX strain was treated with 5 mg/L Er. On the contrary, 2 mg/L of Er decreased the ROS amount by 46.4% and 62.6% in ATCC 43300 and 98825 TX strains respectively, whereas 5 mg/L of the same drug reduced the oxidative stress of 87.1^4^% in the ATCC 43300 biofilm.

### 3.4. Metabolite I of Erdosteine Reduces Biofilm Dispersion

**Figure 5:**
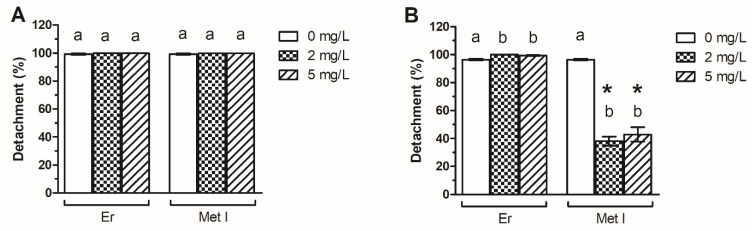
Biofilm dispersion index (%) of MRSA ATCC 43300 (**A**) and 98825 TX (**B**) biofilms pre-grown with Er and Met I at 0, 2 and 5 mg/L and soaked in PBS for 1 h. Data report the mean ± standard deviation of three independent measurements. Different superscript letters indicate significant differences (Tukey’s HSD, *p* ≤ 0.05) between the means of different concentrations whereas a * indicates significant differences between Er and the corresponding counterpart grown with Met I at the same concentration.

ATCC 43300 and 98825 TX biofilms pre-grown with and without Er were almost completely dispersed after a 1-h treatment with PBS (Figure 5 and Supplementary Materials Table S3). The same results were obtained with ATCC 43300 biofilms pre-grown with Met I or Er. On the contrary, significant differences were found between the detachment index of 98825 TX control sample and the same strain grown with Met I. Indeed, 98825 TX biofilms were less prone to be detached by PBS and were more strongly attached onto the surface than the corresponding positive control, with a detachment index corresponding to 60.5% and 55.5% percent when biofilms were pre-grown with Met I at 2 and 5 mg/L respectively.

### 3.5. The 5 mg/L Metabolite I Alters the Expression of ATCC 43300 Biofilm Proteins

nLC-HRMS data performed on ATCC 43300 biofilm proteome are reported in Supplementary Materials Table S4. nLC-HRMS identified 231 proteins in the control sample and 238 proteins in the 5 mg/L Met I treated sample. One protein was identified exclusively in the control sample and 9 proteins were identified exclusively in the treated sample (Supplementary Materials Table S4).

**Figure 6:**
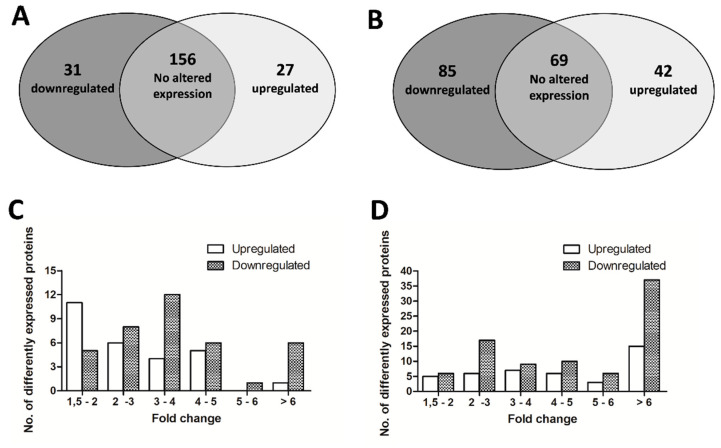
Venn diagram and histogram about proteins differentially expressed in ATCC 43300 (**A**,**C**) and 98825 TX (**B**,**D**) biofilms grown with Met I at 5 mg/L. Panel (**A**,**B**): Venn diagram shows proteins not significantly different in abundance between the positive control and treated samples and differentially abundant proteins with a ±1.5-fold change in the treated biofilm compared to the control one. Panel (**C**,**D**): histograms displays the number of differentially expressed proteins within a specific range of fold changes.

A total of 214 proteins were statistically analysed by *t*-tests in order to evaluate significant differences in abundance between treated and untreated samples (*p* < 0.001). Of the 214 quantified proteins, 156 proteins were not significantly different in abundance between the control and treated samples and 58 proteins, which accounted for nearly 30% of the total proteins, were differentially expressed (±1.5-fold change) in the treated biofilm compared to the control one (Figure 6A, Supplementary Materials Table S5). The highest numbers of biofilm proteins with significant differences were 3–4-fold upregulated and 1.5–2-fold downregulated upon Met I treatment. Only 7 proteins of the treated biofilm showed a 6 or greater fold change in comparison to the control biofilm (6 downregulated and 1 upregulated) (Figure 6C). The volcano plot in Supplementary Materials Figure S4A graphically displays the quantitative data.

To obtain a global view of functions associated with upregulated and downregulated proteins, GO analysis was carried out. A total of 54 of 58 proteins upregulated and downregulated upon Met I treatment were successfully mapped by GO functional analysis according to three main groups generally used to cluster proteins into biologically relevant categories: biological processes, molecular function and cellular compartment [33]. GO terms are reported in Supplementary Materials Table S6. Analysed proteins were assigned to the cytoplasmatic (16 hits) and the plasma membrane compartment (5 hits) (Supplementary Materials Table S6). In terms of molecular processes, upregulated and downregulated proteins were classified mainly in binding (38 hits) of organic cyclic (31 hits) and heterocyclic (31 hits) compounds, ions (23 hits) and small molecules (21 hits). The second most abundant molecular process of differently expressed proteins was catalytic activity (34 hits) including, among others, oxidoreductase (10 hits), transferase (8 hits) and ligase (8 hits) activities. Molecular classification also included structural constituents of ribosomes (8 hits) and transmembrane transporter activity (4 hits) (Supplementary Materials Table S6). Moreover, GO analysis of these proteins revealed that the most frequent of the biological processes was metabolic processes (32 hits) with proteins mostly involved in the metabolism of organic substance (30 hits), including organic acids (15 hits), aromatic compounds (10 hits), carbohydrates (7 hits) and its derivatives (9 hits), as well as nitrogen compounds (26 hits) and small molecules (19 hits). Additionally, proteins were also involved in other cellular processes such as biosynthetic processes (14 hits) as well as regulation (3 hits), transport (2 hits), response to stress (2 hits) and pathogenesis (1 hit) (Supplementary Materials Table S6).

### 3.6. The 5 mg/L Metabolite I Alters the Expression of 98825 TX Biofilm Proteins

nLC-HRMS data performed on the 98825 TX biofilm proteome are reported in Supplementary Materials Table S7. nLC-HRMS analysis allowed the identification of 255 proteins in the control sample and 221 proteins in the 5 mg/L Met I treated sample. A total of 37 proteins were exclusively identified in the control sample and 3 proteins were only present in the treated sample (Supplementary Materials Table S5).

In order to highlight significant differences in abundance between treated and untreated samples (*p* < 0.001), 196 proteins were statistically analysed by *t*-tests. Among these, 69 proteins were not significantly different in abundance between the control and treated samples and 127 proteins, which account for nearly 65% of the total proteins, were differentially expressed (±1.5-fold change) in the treated biofilm compared to the control one (Figure 6B, Supplementary Materials Table S5). The highest numbers of biofilm proteins with significant differences were 6 or greater fold upregulated (15 proteins) or downregulated (37 proteins) in comparison to the control biofilm, while the second highest number of proteins was 2-3-fold downregulated upon the Met I treatment (Figure 6D). The volcano plot in Supplementary Materials Figure S4B,F graphically displays the quantitative data.

A total of 115 of 127 proteins upregulated and downregulated upon Met I treatment were successfully mapped by GO functional analysis. GO terms were reported in Supplementary Materials Table S6. Most of the analysed proteins were classed as cytoplasmatic (33 hits) (Supplementary Materials Table S6). In terms of molecular processes, upregulated and downregulated proteins were classified mainly in catalytic activity (87 hits) with a predominant role of oxidoreductase (23 hits), hydrolase (23 hits) and transferase (19 hits) activity. A relevant portion of differently expressed proteins were also classified as involved in binding activity (85 hits) of heterocyclic (73 hits) and organic cyclic compounds (73 hits), ions (58 hits), small molecules (52 hits) and carbohydrate derivatives (34 hits). Additionally, molecular classification also included proteins that are structural constituents of ribosomes (11 hits) or translation factors (8 hits) and involved in antioxidant activity (2 hits) (Supplementary Materials Table S6). The biological process classification revealed that the most frequent categories of differently expressed proteins were metabolic (67 hits) and cellular processes (66 hits) followed by response to stimulus (6 hits), localization (4 hits) and biological regulation (4 hits). The metabolic category mainly included metabolism of organic substance (59 hits), such as organonitrogen compounds (40 hits), organic acids (25 hits) and carbohydrates (19 hits) and their derivatives (20 hits), metabolism of nitrogen compounds (45 hits) and small molecules (38 hits), catabolic (15 hits) and biosynthetic processes (31 hits). Additionally, proteins were also involved in other biological processes such as response to stimulus (6 hits), including response to oxidative stress (3 hits) and reactive oxygen species (1 hit), localization (4 hits), biological regulation (4 hits) and pathogenesis (1 hit) (Supplementary Materials Table S6).

### 3.7. 5 mg/L Metabolite I Alters the Expression of ATCC 43300 and 98825 TX in a Different Way: Comparison between the Two Strains

Treatment with Met I downregulated 20 proteins and upregulated 8 proteins in biofilms of both bacterial strains (Table 1). Additionally, 10 proteins were differently expressed in both strains but with an opposite trend (Supplementary Materials Table S5). However, 89 proteins were changed in expression only in 98825 TX and 20 proteins were differently expressed only in ATCC 43300 (Supplementary Materials Table S5).

**Figure 7:**
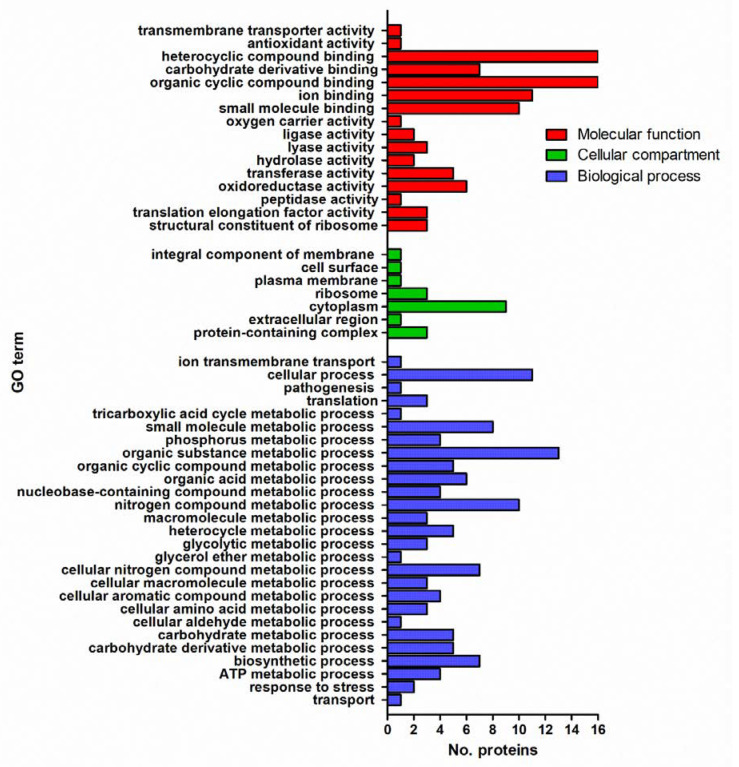
GO functional classification of proteins differently expressed in both ATCC 43300 and 98825 TX biofilms upon Met I at 5 mg/L treatment. GO classification was performed in terms of molecular function (red), cellular compartment (green) and biological process (blue).

The predominant molecular functions from GO classification of these proteins that were found changed with a similar trend in both strains included binding (18 hits) of heterocyclic (16 hits) and organic cyclic (16 hits) compounds as well as ions (11 hits) and small molecules (10 hits). Additionally, proteins with altered expression upon Met I treatment were involved in catalytic activities such as, among others, oxidoreductase activity (6 hits) (Figure 7 and Supplementary Materials Table S6). As regards the biological processes, the same proteins, mainly found in the cytoplasm (9 hits) (Figure 7 and Supplementary Materials Table S6), were involved in primary (12 hits), cellular (11 hits), organic substance (13 hits) and nitrogen compounds (10 hits) metabolic processes. Proteins with a change of expression in both strains also were involved in the regulation of biological process (2 hits), response to oxidative (1 hit) and nitrosative stresses (1 hit), transport (1 hit) and pathogenesis (1 hit) (Figure 7 and Supplementary Materials Table S6).

## 4. Discussion

The effect of Er and Met I on bacterial adhesion, mature biofilm and biofilm dispersal, by setting up a laboratory-scale model system simulating conditions encountered in vivo. Pharmacological concentrations close to the plasma peak value obtained after oral administration and previously used by Braga et al. [26] has been used. Additionally, the impact of ROS on biofilm formation was measured. Notably, the antioxidant properties of Er and Met I are largely documented in human neutrophils and eosinophils [23]. A previous report has shown that the -SH group of Met I is responsible of reducing the amount of N-centred radical species because of a termination reaction between the free radicals and Met I [34]. However, its antioxidant effect on biofilm has never been documented so far.

Data demonstrated that Er and Met I at 2 and 5 mg/L promoted biofilm formation, which is characterized by an increased number biofilm-dwelling cells and a reduced ROS level in comparison to the control samples. It is well known that some antimicrobials at sub-inhibitory concentrations can function as signalling molecules and promote biofilm formation while altering bacterial virulence, quorum sensing, gene expression and gene transfer [35,36,37]. Furthermore, the antioxidant *N*-acetylcysteine promoted biofilm growth in *S. aureus* ATCC25923 and *Pseudomonas aeruginosa* PAO1 by increasing expression of some biofilm-associated genes and enhanced extracellular polymers production [13].

Since 5 mg/L Met I showed the major effect on biofilm development, the proteome of both biofilms grown without and with 5 mg/L Met I was analysed in depth. Comparative proteomics revealed that several proteins, corresponding to 30% and 65% of total proteins in ATCC 43300 and 98825 TX treated strains respectively, were differently expressed, showing a complex interaction of different mechanisms regulating the biofilm behaviour under the antioxidant drug treatment. For 28 proteins, changes were found with a similar trend in both strains, suggesting a key role of these proteins in the response of *S. aureus* to Met I treatment, independently of the strain.

Met I modulated biofilm formation by overexpressing some proteins involved in cells replication. For example, Enolase (Eno), found upregulated in the presence of Met I, is considered a moonlighting protein in the *S. aureus* adhesion. *S. aureus* biofilms secrete this cytoplasmic protein to the outer surface. The protein acts as surface sensing receptor and clumping factor, initiating surface colonization [38,39].

Along the same line, the ATP-dependent Clp protease ATP-binding subunit (ClpL) was found upregulated up to 5.2-fold when the biofilms were grown with Met I. Clp ATPases are a family of closely related proteins required for stress tolerance, intracellular replication and biofilm formation in *S. aureus*. Indeed, they play a critical role in cellular protein quality control systems by refolding or degrading damaged proteins in stressed cells [40]. It was demonstrated that when *clpL* is inactivated in *S. aureus*, the bacterial intracellular multiplication in epithelial cells appeared decreased and cells were significantly smaller [41]. Therefore, it may be possible that ClpL, found overexpressed in the present study, might contribute to enhance the number of biofilm-dwelling cells when Met I is present.

The most upregulated protein was the ATP synthase gamma chain (AtpG). AtpG belongs to the ATP synthase operon, the genes of which encode the subunits of the ATP synthase catalytic core, the central metabolic protein driven by the proton motive force generated by the respiratory chain [42]. With regard to *S. aureus*, a recent study identified that AtpG was needed for virulence in a mouse model of skin and soft tissue infection. When Bosch et al. [42] elicited the *atpA* gene, which is upstream of *atpG* in the operon and, as such, was also inactivated, the growth kinetics under both planktonic and sessile conditions was altered, suggesting its involvement in cellular replication.

The largest group of proteins downregulated in the treated biofilm exploited oxidoreductase activity with mechanisms involved, among others, in the response to oxidative and nitrosative stresses. The treated biofilm displayed a relevant decrease in the amount of ROS. Accordingly, proteins of which the transcription is usually induced by oxidative stress, such as aldehyde dehydrogenase, alkyl hydroperoxide reductase C or thioredoxin, were found downregulated in the treated samples [43,44,45]. Thioredoxin is present in all living cells, including bacteria and fungi, and it acts as an antioxidant system through the conversion of thiol and disulfide bonds [46]. It is a cytoplasmatic protein but it is secreted extracellularly in the matrix in many bacterial biofilms, including *S. aureus*, under infections [47]. Therefore, thioredoxin has a role in *S. aureus* virulence and it is here downregulated.

Nitric oxide (NO) plays a central role as signal molecule in biological pathways. In mammalian hosts, NO is an important mediator produced by immune cells in copious quantities from L-arginine and molecular oxygen. During infection, the high levels of NO produced by immune cells significantly alter microbial metabolism and physiology, in a mechanism called nitrosative stress. These antimicrobial actions help the host by inhibiting pathogen development during infection. In *S. aureus*, NO suppresses bacterial virulence by targeting the Agr quorum sensing system and influencing quorum sensing-mediated bacterial behaviors such as biofilm formation and dispersion [48,49,50]. Interestingly, methicillin resistant *S. aureus* also produces NO, that regulates electron and maintains membrane bioenergetics. This process confers an advantage in terms of virulence and increased sensitivity to oxidative stress and antibiotics [51]. Bacteria sense NO and have evolved diverse ways to cope with nitrosative stress and invalidate host defenses [48]. In *S. aureus*, Hmp represents the main detoxification enzymatic mechanism of radical nitrogen species (RNS), including NO, which are produced by host cells during the immune response to bacterial infection [52]. Hmp expression is strictly controlled by the staphylococcal respiratory regulator (SrrAB), a two-component redox sensor that is implicated, among others, in long-term biofilm stability [53]. Indeed, NO leads to the activation of the SrrAB, which controls expression of *hmp* [54]. This process is essential for *S. aureus* bacterial colonization and biofilm development as well antibiotic and nitrosative stress resistance. Null *hmp* mutants of *Salmonella* and *Escherichia coli* were found defective in virulence and hyper-sensitive to macrophages [51]. Additionally, *S. aureus* adapts to NO by expression of other proteins, e.g., the lactate dehydrogenases and the probable malate:quinone oxidoreductase (Mqo), that maintains the redox balance [55,56]. *S. aureus mqo* mutants displayed a reduction in virulence [57].

Hmp, D-lactate dehydrogenase, and Mqo were found downregulated in both strains and the biofilm was found increased upon Met I treatment. Findings indicated that Met I, a molecule with an SH group, reacts with NO by both an antioxidant and scavenging activity [58]. It is likely that Er, through its metabolite, decreases the NO level within the bacterial cells, deactivating SrrAB and downregulating Hpm and D-lactate in cascade and leading to an increase of biofilm development, maybe with a mechanism involving a quorum sensing pathway. In agreement with our results, *hmp S. aureus* mutants produced higher levels of biofilm in comparison to the wild type [59]. Similarly, low or sub-physiological concentrations of NO were proved to stimulate biofilm formation [60]. Moreover, the AtpG proteins, previously discussed and here found upregulated, are newly identified proteins which are required for growth during NO stress [61].

Accordingly, the membrane-anchored alkaline shock protein 23 (Asp23) mediates the electrostatic interactions with negatively charged surface components, enhancing aggregation and biofilm integrity [62]. This protein was found downregulated, further confirming the Met I activity in weaking the biofilm.

In the present study, the transcriptional regulator SarA (SarA) was found to be downregulated. SarA is a global regulator that controls, in a cell density-dependent manner, the production of *S. aureus* virulence factors that mediate pathogenesis and evasion of the host immune system, including several genes involved in oxidative stress resistance and biofilm formation. Mutations of *sarA* in different *S. aureus* strains influenced the regulation of biofilm by decreasing the production of the polysaccharide intercellular adhesin (PIA), the dominant part of the extracellular matrix [63,64]. SarA also acts as a transcriptional activator of the genes for fibronectin binding proteins (Fnb). Indeed, the expression of both proteins was inhibited in the *sarA* mutants [65]. Fnb proteins are thought to be important for the adhesion and enhance platelet aggregation of *S. aureus* during infection [66]. Accordingly, fnb mutants lacked the ability to adhere. As SarA is downregulated by Met I, a reduction in the content of PIA and Fnb proteins in the treated biofilm is to be expected.

EsxA is secreted by the ESAT-6-like system that is required for the pathogenesis of *S. aureus* infections [67]. In the current study, the ESAT-6-like protein was found downregulated in both treated biofilms with the highest fold change values. Mutants failing to secrete EsxA showed defects in the pathogenesis of *S. aureus* murine abscesses, suggesting that this specialized secretion system may be a general strategy of human bacterial pathogenesis [68].

The ribosomal proteins 30S and 50S are also considered non classical proteins relevant to virulence and resistance and were found here downregulated when biofilms were grown in the presence of Met I.

Overall, our research demonstrated that Met I is able to reduce bacterial virulence, beside increasing the number of sessile cells during the drug treatment. This effect is promising in enhancing the efficacy of antibiotics treatments. Indeed, once dispersed from the biofilm and lacking in virulence, free-floating bacteria are more susceptible to antibiotic treatments [69]. In line with this consideration, Er has shown a synergism with antibiotic therapies, potentiating the inhibition of *S. aureus* in comparison to the antibiotic alone and significantly improving the outcome of chronic obstructive pulmonary diseases [2,24,70,71,72].

## 5. Conclusions

This research provides new science-based evidence to elucidate the role of Er and Met I in biofilm formation. Here we found that Er and Met I increased the number of biofilm-dwelling cells, probably as a consequence of upregulation of some proteins involved in cells replication. Additionally, a reduction of NO concentration is assumed to increase the biofilm development through a mechanism involving the modulation of quorum sensitive pathways. Several proteins normally involved in virulence were here downregulated in the presence of Met I, suggesting that treated cells, despite greater in number, might have lost part of their virulence. Therefore, Er and metabolite have the potential to enhance the antibiotic action and to act as adjuvants of the human immune response when an infection is present. Further studies could confirm this potential.

The results reported here will add information towards reaching a more complete comprehension about the biological processes of biofilms upon administration of Er, information useful for the improvement of clinical practices and therapeutic modalities.

## Figures and Tables

**Table 1:** Proteins differently expressed in both ATCC 43300 and 98825 TX biofilms upon Met I treatment. Ratio Met I/Control was calculated by dividing the LFQ mean intensity value of the treated samples by that of controls.

Accession	Protein Name	Ratio: Met I/Control	
**Upregulated in Both Strains**		**ATCC 43300**	**98825 TX**
A0A6B5GER5	ATP synthase gamma chain	4.115	3.731
A0A5C8XBW5	ATP-dependent Clp protease ATP-binding subunit	3.247	5.263
A0A1Q8DC93	Phosphoglycerate kinase	2.778	5.780
A0A6B0BUI5	Enolase	2.075	2.262
A0A2S6DJ38	30S ribosomal protein S4	1.815	1.565
A0A5C8X8W0	Ornithine carbamoyltransferase	1.669	21.739
A0A1Q8DBU3	30S ribosomal protein S3	1.536	1.770
A0A2S6DKC3	Elongation factor G	1.502	4.630
**Downregulated in Both Strains**			
A0A0E1X760	Ornithine aminotransferase	0.646	0.178
A0A077UHY4	Elongation factor Ts	0.589	0.507
A0A5F0TDJ0	Fructose-bisphosphate aldolase class 1	0.573	0.377
A0A0E0VNI4	Glutamine synthetase	0.424	0.181
Q2YSV9	Transcriptional regulator SarA	0.391	0.184
T1YCP4	Aldehyde dehydrogenase	0.389	0.372
A0A0E1VIR3	Pyruvate kinase	0.382	0.539
T1Y9F1	Transcription elongation factor GreA	0.321	0.144
A0A0E0VNW0	Thioredoxin	0.279	0.140
A0A0E1VGN7	Universal stress protein	0.267	0.059
A0A0E0VU32	D-lactate dehydrogenase	0.257	0.337
T1YCX2	Probable malate:quinone oxidoreductase	0.235	0.089
A0A0E1VJH6	30S ribosomal protein S1	0.217	0.055
A0A077ULP0	Alkaline shock protein 23	0.200	0.050
A0A0E0VL26	Alkyl hydroperoxide reductase C	0.167	0.044
A0A0E1XIM1	Glutamine-ructose-6-phosphate aminotransferase	0.158	0.161
A0A380DHX1	Flavohemoglobin	0.157	0.191
A0A6B0BAU5	Ketose-bisphosphate aldolase	0.122	0.074
A0A077W3A7	50S ribosomal protein L18	0.043	0.208
A0A5C8X621	ESAT-6-like protein	0.039	0.055

## Data Availability

The data presented in this study are available in Supplementary Materials.

## Supplementary Materials

The following are available online at https://www.mdpi.com/article/10.3390/antiox10121922/s1, Figure S1: MRSA planktonic growth with Er and Met I at 0, 2 and 5 mg/L, Figure S2. Representative views of 3-D CLSM images of biofilm grown without (A, D) and with Er (B, E) and Met I (C, F) at 5 mg/L. Live cells bacteria were stained green with SYTO 9 fluorescent nucleic acid dye. Scale bar = 50 or 100 μm, Figure S3. Representative views of 3-D CLSM images of biofilm grown without (A, D) and with Er (B, E) and Met I (C, F) at 5 mg/L. The biofilm matrix was visualized in red by the lectin Concanavalin A stain. Scale bar = 50 or 100 μm, Figure S4. Volcano plot about proteins differentially expressed in ATCC 43300 (A) and 98825 TX (B) biofilms grown with Met I at 5 mg/L. The −log10 (p value) is plotted against the log2 (fold change: Met I/Control). Points above the non-axial horizontal line represent proteins with significantly different abundances (*p* < 0.001). Points to the left of the left-most non-axial vertical line denote protein fold changes of Met I/Control less than −1.5, while points to the right of the right-most non-axial vertical line denote protein fold changes of Met I/Control greater than 1.5, Table S1: Adhered cells (CFU/cm^2^) within MRSA ATCC 43300 and 98825 TX biofilms grown with Er and Met I at 0, 2 and 5 mg/L, Table S2: percentage reduction in the oxidative stress level within the MRSA ATCC 43300 and 98825 TX biofilms grown with Er and Met I at 0, 2 and 5 mg/L, Table S3: biofilm dispersion index (%) of MRSA ATCC 43300 and 98825 TX biofilms pre-grown with Er and Met I at 0, 2 and 5 mg/L and soaked in PBS for 1 h, Table S4: nLC-HRMS analysis of proteins extracted from the ATCC 43300 biofilm grown without and with Met I at 5 mg/L, Table S5: proteins differently expressed in ATCC 43300 and 98825 TX biofilms upon Met I treatment, Table S6: GO functional classification of proteins significantly upregulated and downregulated in ATCC 43300 and 98825 TX biofilms upon Met I at 5 mg/L treatment, Table S7: nLC-HRMS analysis of proteins extracted from the 98825 TX biofilm grown without and with Met I at 5 mg/L.
