# Supplementary material for: Understanding the Role of the Antioxidant Drug Erdosteine and Its Active Metabolite on Staphylococcus aureus Methicillin Resistant Biofilm Formation
Source: Antioxidants (Basel). 2021 Nov 29;10(12):1922. doi: 10.3390/antiox10121922 (PMC8698571; doi:10.3390/antiox10121922)
Supplement: Supplementary file 1 [file antioxidants-10-01922-s001.zip › Supplementary materials.pdf]

Supplementary materials

Understanding the role of the antioxidant drug Erdosteine and its active metabolite on *Staphylococcus aureus* methicillin resistant biofilm formation

Cristina Cattò*, Federica Villa, Francesca Cappitelli
[a]*Department of Food Environmental and Nutritional Sciences, Università degli Studi di Milano, Milano, Italy*

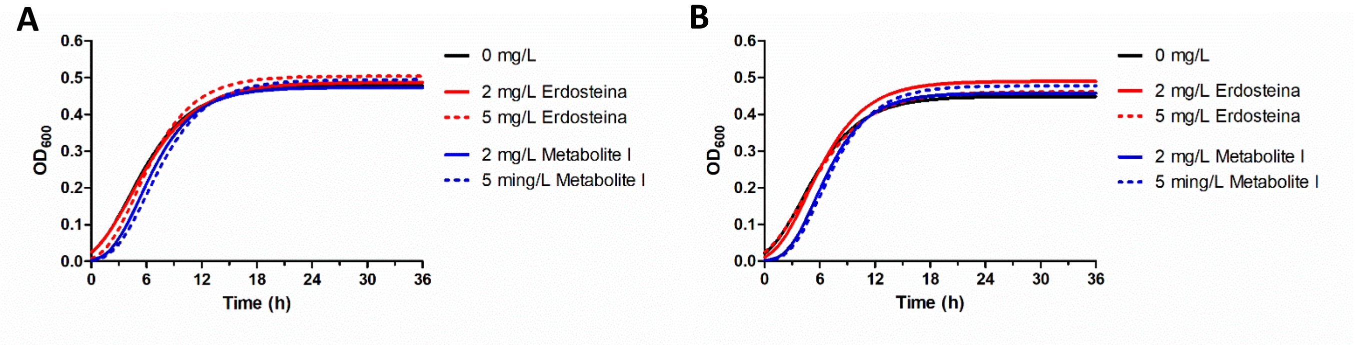

C

			0 mg/L	2 mg/L	5 mg/L
ATCC 43300	Erdosteine	μ_m (A_{600}/h × 10^{-2})	4.81±0.27	4.57±0.50	4.62±0.50
		Y_M (A_{600} × 10^{-1})	4.63±0.18	4.86±0.19	4.89±0.22
		R^2	0.989±0.004	0.990±0.002	0.992±0.004
	Metabolite I	μ_m (A_{600}/h × 10^{-2})	4.81±0.27	5.25±0.20	5.02±0.20
		Y_M (A_{600} × 10^{-1})	4.63±0.18	4.77±0.22	4.75±0.07
		R^2	0.989±0.004	0.996±0.002	0.995±0.003
98825 TX	Erdosteine	μ_m (A_{600}/h × 10^{-2})	4.60±0.20	4.62±0.10	4.35±0.20
		Y_M (A_{600} × 10^{-1})	4.65±0.13	4.74±0.12	4.89±0.15
		R^2	0.990±0.004	0.990±0.005	0.987±0.005
	Metabolite I	μ_m (A_{600}/h × 10^{-2})	4.60±0.20	4.99±0.20	4.99±0.23
		Y_M (A_{600} × 10^{-1})	4.65±0.13	4.62±0.12	0.47±0.15
		R^2	0.990±0.004	0.993±0.006	0.993±0.006

Figure S1. MRSA planktonic growth with Er and Met I at 0, 2 and 5 mg/L. Panel A displays OD_{600}-based growth curves whereas in panel B growth parameters of maximum specific growth rate (μ_m), maximum growth and the Goodness of Fit (R^2) obtained by the Gompertz model are reported. Data represent the mean ± standard deviation of three independent measurements. No statistical differences were found.

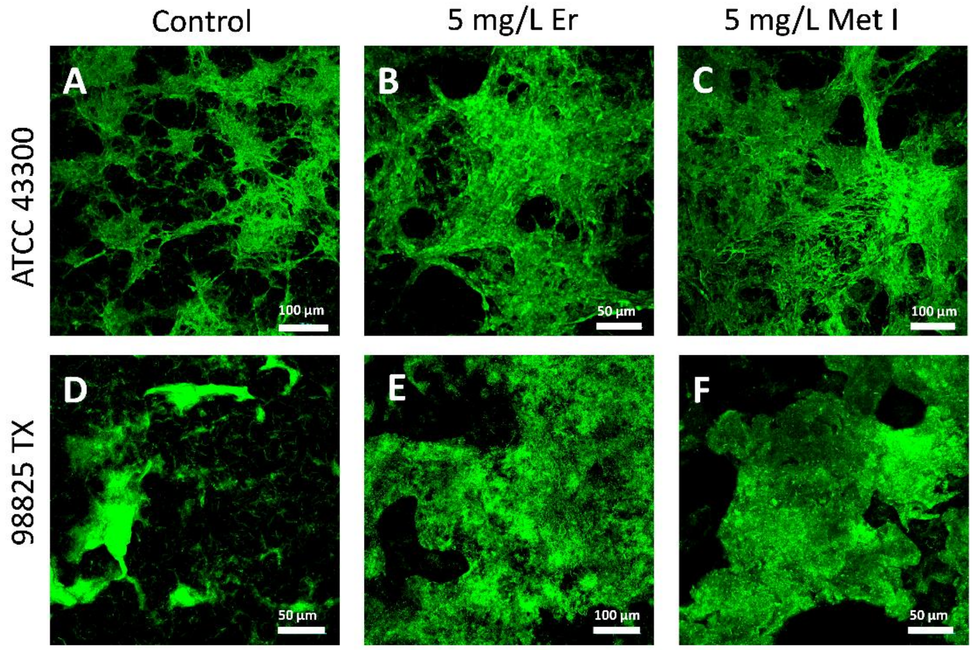

Figure S2. Representative views of 3-D CLSM images of biofilm grown without (A, D) and with Er (B, E) and Met I (C, F) at 5 mg/L. Live cells bacteria were stained green with SYTO 9 fluorescent nucleic acid dye. Scale bar=50 or 100 µm.

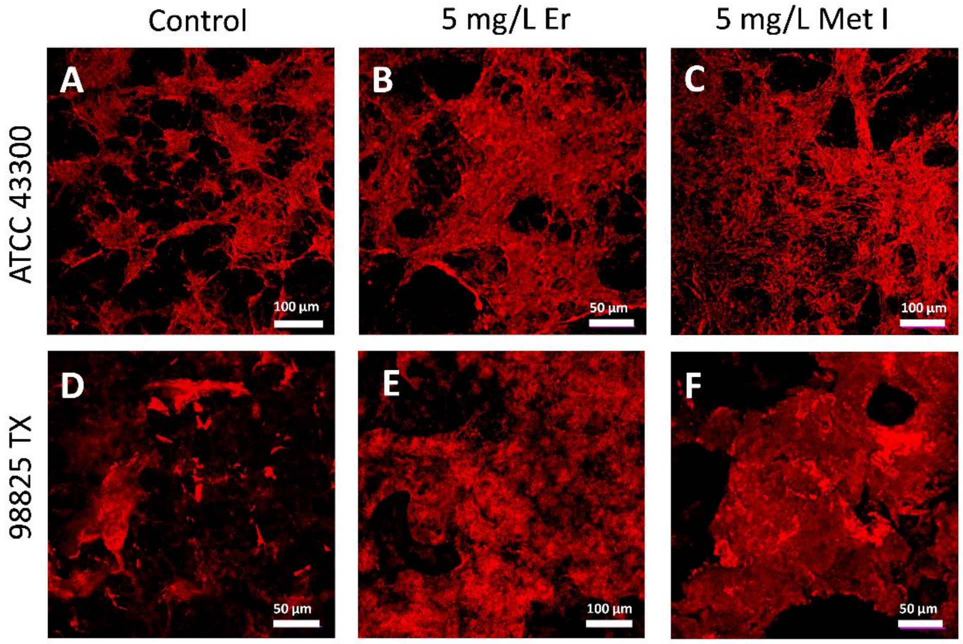

Figure S3. Representative views of 3-D CLSM images of biofilm grown without (A, D) and with Er (B, E) and Met I (C, F) at 5 mg/L. The biofilm matrix was visualized in red by the lectin Concanavalin A stain. Scale bar=50 or 100 µm.

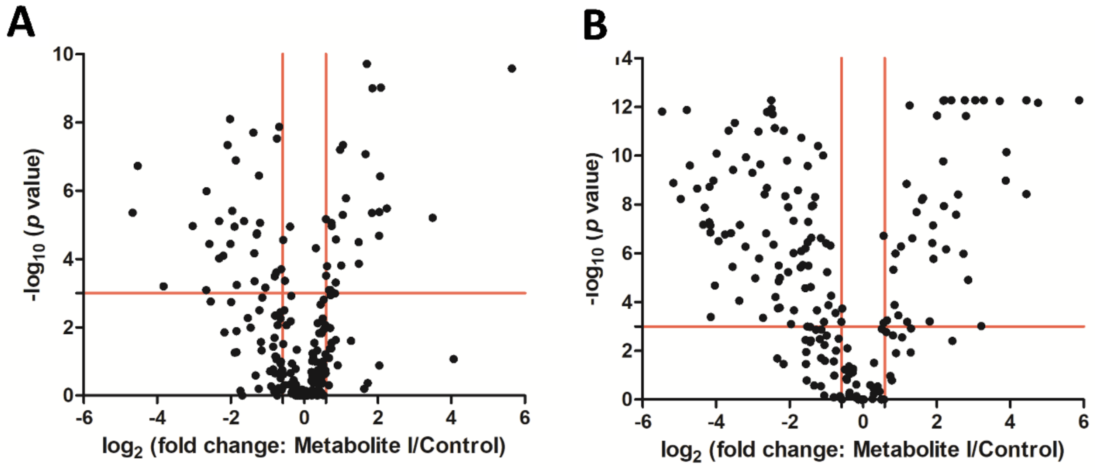

Figure S4. Volcano plot about proteins differentially expressed in ATCC 43300 (A) and 98825 TX (B) biofilms grown with Met I at 5 mg/L. The $-\log_{10}$ (p value) is plotted against the $\log_2$ (fold change: Met I/Control). Points above the non-axial horizontal line represent proteins with significantly different abundances ($p < 0.001$). Points to the left of the left-most non-axial vertical line denote protein fold changes of Met I/Control less than - 1.5, while points to the right of the right-most non-axial vertical line denote protein fold changes of Met I/Control greater than 1.5.

		0 mg/L	2 mg/L	5 mg/L
**ATCC 43300**	**Erdosteine**	$(4.05\pm0.84)\times10^{9\ a}$	$(7.01\pm2.39)\times10^{9\ b}$ [+ (0.73±0.25) fold]	$(2.56\pm0.72)\times10^{10\ c}$ [+ (5.32±1.49) fold]
	**Metabolite I**	$(4.05\pm0.84)\times10^{9\ a}$	$(2.80\pm0.72)\times10^{11\ b\,*}$ [+ $(6.81\pm1.75)\times10$ fold]	$(4.71\pm0.63)\times10^{11\ c\,*}$ [+ $(1.15\pm0.15)\times10^{2}$ fold]
**98825 TX**	**Erdosteine**	$(1.20\pm0.26)\times10^{8\ a\,\bullet}$	$(2.00\pm0.25)\times10^{8\ b\,\bullet}$ [+ (0.67±0.08) fold]	$(1.06\pm0.25)\times10^{10\ c\,\bullet}$ [+ $(8.71\pm2.04)\times10$ fold]
	**Metabolite I**	$(1.20\pm0.26)\times10^{8\ \bullet}$	$(6.18\pm0.58)\times10^{11\ b\,*\,\bullet}$ [+ $(5.15\pm0.48)\times10^{3}$ fold]	$(9.14\pm1.32)\times10^{11\ c\,*\,\bullet}$ [+ $(7.61\pm1.10)\times10^{3}$ fold]

Table S1. Adhered cells (CFU/cm^2) within MRSA ATCC 43300 and 98825 TX biofilms grown with Er and Met I at 0, 2 and 5 mg/L. In the brackets, increase in comparison to the control sample (0 mg/L) is reported. Data represent the mean ± standard deviation of three independent measurements. Different superscript letters indicate significant differences (Tukey's HSD, $p \leq 0.05$) between the means of different concentrations, a star indicates significant differences between Er and the corresponding counterpart grown with Met I at the same concentration whereas a dot indicates significant differences between ATCC 43300 and 98825 TX treated with the same drug and concentration.

		0 mg/L	2 mg/L	5 mg/L
**ATCC 43300**	**Erdosteine**	-	$46.44\pm3.55^{\ a}$	$87.10\pm5.10^{\ b}$
	**Metabolite I**	-	$99.76\pm2.47^{\ a\,*}$	$99.16\pm1.07^{\ a\,*}$
**98825 TX**	**Erdosteine**	-	$62.65\pm0.85^{\ a\,\bullet}$	$98.72\pm0.71^{\ b\,\bullet}$
	**Metabolite I**	-	$99.94\pm1.50^{\ a\,*}$	$99.96\pm0.28^{\ a}$

Table S2. Percentage reduction in the oxidative stress level within the MRSA ATCC 43300 and 98825 TX biofilms grown with Er and Met I at 0, 2 and 5 mg/L. Data represent the mean ± standard deviation of three independent measurements. Different superscript letters indicate significant differences (Tukey's HSD, $p \leq 0.05$) between the means of different concentrations, a star indicates significant differences between Er and the corresponding counterpart grown with its Met I at the same concentration, whereas a dot indicates significant differences between ATCC 43300 and 98825 TX treated with the same drug and concentration.

		0 mg/L	2 mg/L	5 mg/L
**ATCC 43300**	**Erdosteine**	$99.96\pm0.58^{\ a}$	$99.98\pm0.01^{\ a}$	$99.97\pm0.02^{\ a}$
	**Metabolite I**	$99.96\pm0.58^{\ a}$	$99.86\pm0.01^{\ a}$	$99.84\pm0.05^{\ a}$
**98825 TX**	**Erdosteine**	$94.71\pm2.44^{\ a\,\bullet}$	$99.92\pm0.03^{\ b}$	$99.36\pm0.26^{\ b}$
	**Metabolite I**	$94.71\pm2.44^{\ a\,\bullet}$	$38.09\pm3.27^{\ b\,*\,\bullet}$	$42.89\pm5.12^{\ b\,*\,\bullet}$

Table S3. Biofilm dispersion index (%) of MRSA ATCC 43300 and 98825 TX biofilms pre-grown with Er and Met I at 0, 2 and 5 mg/L and soaked in PBS for 1 h. Data represent the mean ± standard deviation of three independent measurements. Different superscript letters indicate significant differences (Tukey's HSD, $p \leq 0.05$) between the means of different concentrations, a star indicates significant differences between Er and the corresponding counterpart grown with its Met I at the same concentration, whereas a dot indicates significant differences between ATCC 43300 and 98825 TX treated with the same drug and concentration.
